# Alzheimer’s Disease Prediction and Implications for Insurance

**DOI:** 10.1002/alz.088920

**Published:** 2025-01-09

**Authors:** Julia Wittrock, Björn Schmitz‐Luhn

**Affiliations:** ^1^ Center for Life Ethics, University of Bonn, Bonn Germany

## Abstract

**Background:**

The possibilities to determine the individual risk of persons to develop future Alzheimer’s Disease and dementia have greatly increased in recent years. Promising and little invasive methods, e.g. blood or urine tests, may soon be available as predictive or early‐detection measures even for the general population. However, the knowledge incurred may be of interest not only for the affected persons themselves, but also for third parties. Particularly, the results of predictive tests may prompt disclosure or notification obligations to insurers, in turn affecting availability or affordable premiums for life or health insurance plans. This can be a substantial obstacle and burden for individuals considering to undergo predictive diagnosis. As predictive methods become more readily available, the problem gains pressing practical importance.

**Method:**

We analyze existing legislation and jurisprudence in a variety of jurisdictions to investigate what regulations are in place for dealing with individual risk probabilities, and to what extent these are applicable or transferable to the particular problematic aspects regarding the prediction of Alzheimer’s Disease and/or dementia.

**Result:**

Existing insurance law, where in place, strikes only a rudimentary balance, and for a limited scope, mostly genetic diagnostics only. At the same time, access to disease prediction by non‐genetic means is quickly growing, and legal protections, rights and duties are largely unregulated in spite of a similar conflict of interests. The associated uncertainties for the involved parties keep increasing, and there is a pressing need for rules bringing the conflicting interests into a fair balance.

**Conclusion:**

We present efforts and their shortcomings to reduce the tension between the interest of insurers in offering affordable and calculable premiums on the basis of individual risk factors, and the particular need of affected persons to protect the results of predictive examinations due to their high sensitivity. We show how this tension could be sensibly resolved by taking into account ethical and societal values, considering the complex interests at stake.

